# Oral and Maxillofacial Injuries in Civilian Recruits During Mandatory Combat Training at Military Garrisons: A Nationwide Survey

**DOI:** 10.5812/traumamon.6982

**Published:** 2012-10-10

**Authors:** Mohammad Hosein Kalantar Motamedi, Ali Ebrahimi, Amin Askary

**Affiliations:** 1 Trauma Research Center, Baqiyatallah University of Medical Sciences, Tehran, IR Iran

**Keywords:** Military, Training, Wounds and Injuries

## Abstract

**Background:**

There is significant prevalence of physical injuries sustained by civilian recruits at military training garrisons. Civilian recruits sustain these injuries mostly during the intensive and rigorous military combat-training period.

**Objectives:**

We sought to determine the prevalence and causes of oral and maxillofacial injuries as the first step in reducing and preventing them in civilian recruits (males aged over18 years) during their 2-year mandatory military service.

**Materials and Methods:**

In this 2-year study, we referred to 11major military training garrisons in 8 provinces and collected data from available medical records of military clinics at each garrison. Injuries occurring in civilian recruits during the intense 2-month military combat training period were documented. Data regarding the number of civilian trainees, percentage of those injured, site where the injury was sustained, type of injury and its causes, etc. as well as demographic data were collected.

**Results:**

The number of civilians called to military service was 153, 886. The ratio of those injured was 4419/153,886. The percentage of maxillofacial injuries was 20.4% (903/4419). The majorities of maxillofacial injuries occurred during the first month (38%) and were due to nonmilitary (86%) rather than military (14%) causes. From among the military causes, bullets (66%) were the most common cause of injury, while falls (73%) were the major cause of nonmilitary injuries. Mountainous terrain was the main cause of falls (51%). The most common military incidents which led to injury were related to artillery fire and explosions (33%). Nasal bone fracture was the most common maxillofacial fracture (49%), and lacerations were the most common soft tissue injury (54%). Among dental injuries, tooth fracture was most common (66%).

**Conclusions:**

The large number of general and maxillofacial injuries in civilian recruits during the 2-month combat-training period at military garrisons is disconcerting. This issue warrants further research to implement methods for identifying, decreasing, and preventing injuries in civilians at military-training garrisons.

## 1. Background

Trauma is an important health issue both in the civilian population and in military personnel. These injuries represent a continuum of severity from minor injuries to those resulting in lost workdays, long-term disability, and fatalities (1). The prevalence of physical injuries occurring in civilian recruits at military training garrisons is significant and warrants assessment because they are not military personnel but rather recruits who must return to civil life after completion of the country’s 2-year mandatory military service (MMS); they comprise high school graduates as well as those with higher education, e.g., doctors, dentists, engineers, pharmacists, architects, who if disabled may not be able to return to normal civilian life after MMS.

## 2. Objectives

The objective of the present study was to determine the number of injuries, type of oral-maxillofacial (OMF) trauma, causes (military or nonmilitary), the type of training leading to injury, site of injury, and the time period during which the injury was sustained. This was done in order to call for preventive methods, decrease morbidity and reduce the incidence of military trauma in this population of civilian recruits.

## 3. Materials and Methods

In this descriptive retrospective 2-year study, we referred to the clinics of 11 major military training garrisons in 8 provinces and collected data from medical records of injured civilian trainees. Injuries occurring during the intense 2-month military combat-training period were documented. Data regarding the total number of trainees (during the 2-year period), percentage of those injured (all bodily injuries), site where injury was sustained (barracks, battle field, training camp, etc.), percentage and type of maxillofacial injury (soft tissue injury, hard tissue injury, dental trauma), the cause of injury (military or nonmilitary), and type of incident (falling from bunk beds or while jumping hurdles or running, bullets, explosions, etc.) were evaluated. The most common time period within this 2-month military combat-training period wherein most injuries occurred was also noted.

## 4. Results

The number of civilians called to military service in 2009 and 2010 at 11 garrisons was 153,886. The ratio of those injured was 4419/153,886. Nasal bone fracture was the most common maxillofacial fracture (49%, 95% confidence interval [CI] 0.44-0.54), and lacerations were the most common soft tissue injury (54%, 95% CI 0.51-0.57). Among dental injuries, tooth fracture had the highest prevalence (66%, 95% CI0.58-0.73).

### 4.1. Prevalence

Based on the data, the prevalence of OMF injuries was 20.4% (903/4419). The most common time period (first month, second month, last 3-day military camp) within the 2-month military combat-training period when most injuries occurred was during the first month 343/903 (38%, 95% CI 0.35-0.42), 309/903 (34%) of traumas occurred during the final 3 day military camp, and the second month was the least common period during which injuries occurred which comprised 245/903 (28%).

### 4.2. Causes

**Figure 1:**
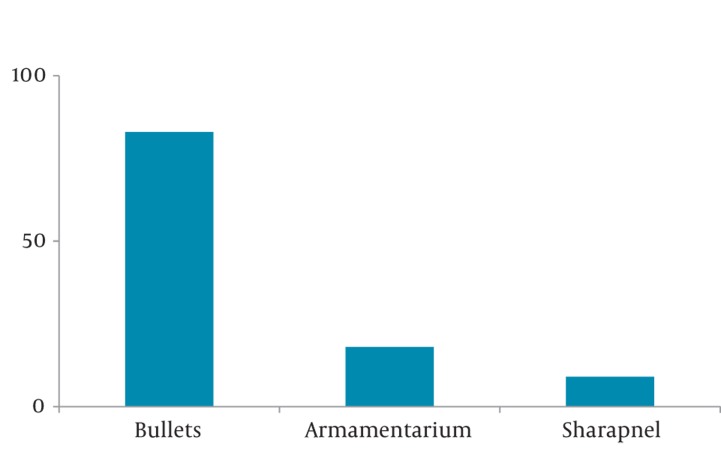
Distribution of Military Causes of Oral and Maxillofacial Injuries

**Figure 2:**
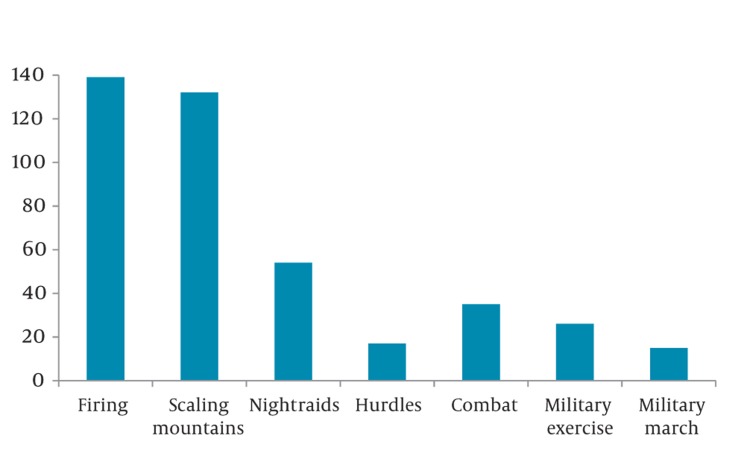
Types of Military Training Leading to Oral and Maxillofacial Injury

The majority of maxillofacial injuries were due to nonmilitary (86%, 95% CI 0.84-0.88) rather than military (14%) causes. Among nonmilitary causes, falls were a major cause of injuries (73%, 95% CI 0.70_0.76). Mountainous terrain was the main cause of falling (51%, 95% CI 0.47-0.55). Among military causes, bullets were the most common (66%, 95% CI 0.58-0.74; Figure 1). The most common military training that led to injury related to artillery fire and explosions (33%, 95% CI 0.41-0.58; Figure 2).

### 4.3. Maxillofacial Injuries

**Figure 3:**
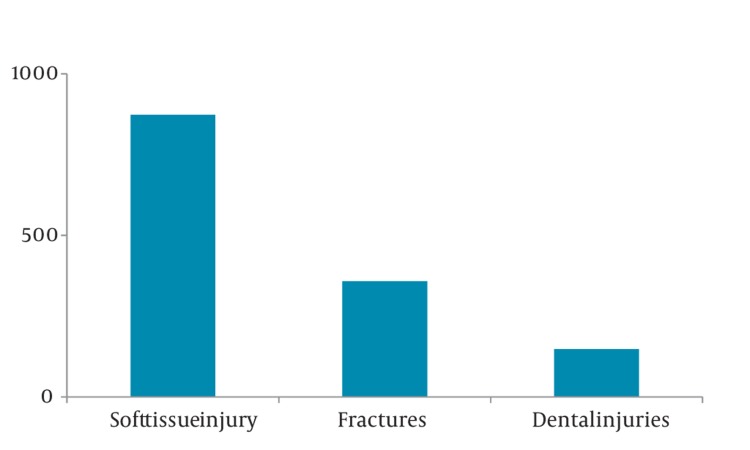
Distribution of Oral and Maxillofacial Injuries Sustained

**Figure 4:**
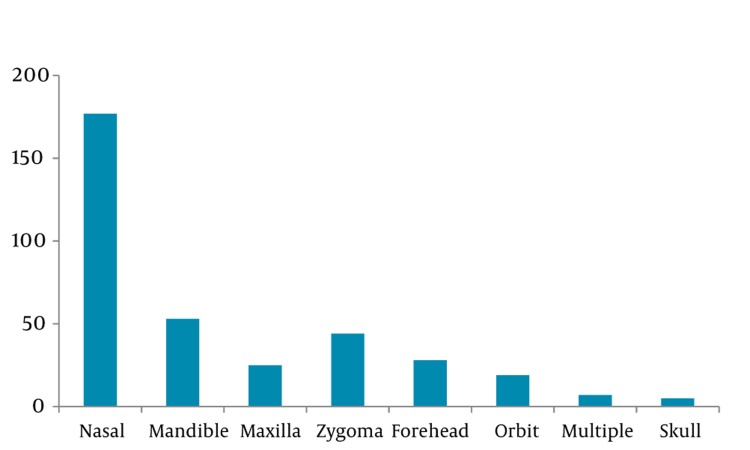
Distribution of Facial Fractures

**Figure 5:**
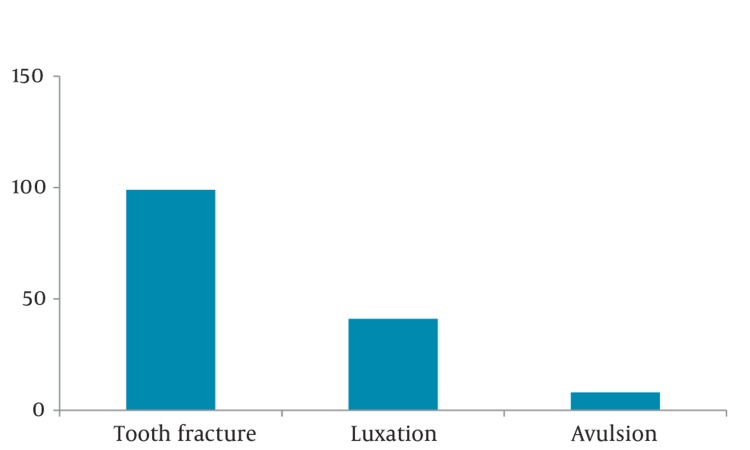
Type of Dental Injuries

96% of cases suffered soft tissue injuries, and 39% of them had bone fractures as well. Incidence of dental trauma was 16%, and eye injury was 7% (Figure 3). Nasal fracture was the most common maxillofacial fracture (49%, 95% CI 0.44-0.54; Figure 4) followed by mandibular fractures (15%). Laceration was the most common soft tissue injury (54%, 95% CI 0.51-0.57), and the percentage of contusion and abrasion was 46%. Among dental injuries (tooth fracture, tooth luxation, avulsion), tooth fracture had the highest percentage (66%, 95% CI 0.58-0.74; Figure 5).

## 5. Discussion

In IR Iran, all men aged above 18 years are required to serve the 2-year term of mandatory military service unless they have disability precluding this or are otherwise exempt. Most injuries occur during the intense 2-month military orientation and combat-training period. According to our findings the prevalence of maxillofacial injuries in civilian recruits was 20.4% (903/4419). The major cause of injury was nonmilitary and therefore may be preventable. Rudzki reported a prevalence of 37.7% in Australian recruits (2). He reported the major cause of nonmilitary trauma in Australian recruits to be altercations (30%), sports (20%), and motor vehicles (15%). Based on Najafi et al.’s study, the most common causes of trauma in military training garrisons were falls (49%) and sports (26.6%) (3). Falls occurred more often in rigorous physical exercises, such as man-to-man combat, jumping hurdles, escalating barriers, and scaling mountains. Motamedi et al. reviewed trauma patient records in hospitalized trauma patients and noticed that the most common causes of traumas after traffic accidents were falls (27%), bullets and shrapnel (6%), and altercations (11%) (4). However, these included all hospitalized trauma patients (civilian and military), not only recruits.

In our evaluation, 96% of cases suffered from soft tissue injury and 39% suffered bone fractures and 16% dental trauma. Nasal bone fracture was the most common maxillofacial fracture (49%) followed by mandibular fractures (15%). In Jones et al.’s study, the most common fractures were nasal (37.7%) and mandibular (30%) (5). In our study, 16% of OMF injuries were dental injuries (tooth fracture, tooth luxation, avulsion), and the most common type was tooth fracture (66%). According to Becker and Ashkenazi’s study, 1.6% of general traumas in military training garrisons were dental injuries, and the most common type was tooth fracture (90%); falls were the main cause in 15.7% (6). In Levin et al.’s study, the percentage of dental injuries in maxillofacial trauma was 25% (7). According to Breeze et al.’s study, the most common type of maxillofacial injury was laceration, followed by maxillary fracture (8). In our study, laceration (54%) and contusion/abrasion (46%) were the most common soft tissue injuries. The large number of general and OMF injuries in civilians during the 2-month combat training period at military garrisons is disconcerting although the rate is almost the same as many similar studies. This issue warrants further research to implement methods for preventing and decreasing injuries sustained at military-training garrisons.
